# Medium-Term Visual Outcomes of Apodized Diffractive Multifocal Intraocular Lens with +3.00 D Addition Power

**DOI:** 10.1155/2014/247829

**Published:** 2014-03-03

**Authors:** Xiaohong Guo, Yi Sun, Bowen Zhang, Danying Zheng

**Affiliations:** ^1^Cataract Division 1, State Key Laboratory of Ophthalmology, Zhongshan Ophthalmic Center, Sun Yat-Sen University, No. 54 South Xianlie Road, Guangzhou 510060, China; ^2^Department of Ophthalmology, Henan Provincial People's Hospital, Zhengzhou 450003, China; ^3^Department of Ophthalmology, Third Affiliated Hospital of Sun Yat-Sen University, Guangzhou 510630, China; ^4^Surgical Department, The First Affiliated Hospital of Guangzhou Medical University, Guangzhou 510120, China

## Abstract

*Purpose*. To evaluate 2-year visual acuities and questionnaire after bilateral implantation of SN6AD1 multifocal intraocular lens (MIOL) or SN60WF IOL. *Methods*. Patients randomly scheduled for bilateral implantation of SN6AD1 MIOL and SN60WF IOL with 2-year follow-up were enrolled. Uncorrected/corrected distance and near visual acuity, uncorrected intermediate visual acuity at 63 cm under high and low contrast, reading activity, the defocus curve, and a quality-of-life questionnaire were evaluated. *Results*. Each group comprised 20 patients. Uncorrected intermediate visual acuities and uncorrected near visual acuity were better in SN6AD1 group than in SN60WF group (*P* = 0.005, *P* = 0.011, and *P* < 0.001). In SN6AD1 group, the uncorrected intermediate and near visual acuities 1 year and 2 years postoperatively were reduced than postoperative 3-month outcomes, respectively. SN6AD1 group reported superior overall spectacle independence and inferior satisfaction. SN6AD1 group had a longer reading newspaper duration than SN60WF group (*P* = 0.036). When using mobile phone, SN6AD1 group had a more comfortable distance than SN60WF group (*P* < 0.001) and higher speed of reading fixed text message (*P* < 0.001). *Conclusion*. SN6AD1 MIOL provided a satisfactory full range of visual acuities and questionnaire performance 2 years postoperatively. One-year and 2-year uncorrected near and intermediate visual acuities of SN6AD1 MIOL were lower than those 3 months postoperatively.

## 1. Introduction

Multifocal intraocular lens (MIOL) is considered a prevailing alternative to restore the functional vision from far to near independent of glasses. Many clinical studies on diffractive MIOLs [1–3], refractive MIOLs [4–6], or hybrid MIOLs [7–11] in enhancing quality of vision showed promising outcomes. AcrySof ReSTOR SN6AD1 MIOL was developed in 2008. So far, studies have confirmed the satisfactory visual outcomes of SN6AD1 MIOL over a short period [12–15]. In our previous study, we found SN6AD1 MIOL provided a full range of functional visual acuity, reading ability, and high patient satisfaction in spite of a relatively more undesired visual disturbance [16–18]. However, to the best of our knowledge, no medium-term or long-term studies of this type of MIOL are available.

The purpose of this study was to assess the visual performance 2 years after cataract surgery with bilateral implantation of SN6AD1 MIOL. SN60WF IOL, being of the same material and aspherical optic design, was used as the control.

## 2. Material and Methods 

The prospective, random study was approved by the ethics committee of our hospitals and adhered to the tenets of the Declaration of Helsinki. Informed consent was obtained from all patients after the nature and possible consequences of the study were explained. Forty consecutive patients (80 eyes) who had sequential bilateral cataract extraction and IOL implantation from January 2011 to May 2011 were included in this study. SN6AD1 group and SN60WF group were divided according to the types of IOL implanted. Patient selection was continued until 20 patients scheduled for multifocal SN6AD1 IOL implantation and 20 patients scheduled for SN60WF IOL implantation were enrolled.

The inclusion criteria were age between 50 and 78 years, corneal preoperative astigmatism less than 1.0 D, and availability for postoperative examinations. Exclusion criteria included diseases other than cataract (severe systemic diseases, amblyopia, corneal diseases, uveitis, retinopathy, or glaucoma), history of ocular surgery, and astigmatism greater than 1.0 D. The intraoperative exclusion criteria were significant vitreous loss with inability of in-the-bag IOL implantation and anterior chamber hyphema.

The target refraction was −0.25 D to +0.25 D for both IOL groups. The SRK-T or Haigis formula was used for IOL power calculations according to the axis length.

### 2.1. Surgical Technique

All surgeries were performed by only one experienced surgeon using phacoemulsification with the Infiniti Vision System (Alcon). After topical anaesthesia with Alcaine 0.05% and a temporal 3.0 mm clear corneal incision, a central continuous curvilinear capsulorhexis approximately 5.5 mm in diameter was created. Phacoemulsification with torsional ultrasound was followed by irrigation and aspiration of the cortex and IOL implantation in the capsular bag using a Monarch II injector (Alcon, Inc.). The position of IOL postoperatively was detected by slit-limp microscopy imaging system with maximum pupil dilation. Anterior Segment Optical Coherence Tomography will be needed if necessary.

### 2.2. Main Outcome Measures

All patients had examinations over a 2-year follow-up period after surgery. Postoperative evaluations were performed at 1 day, 1, 3, and 6 months, and 1 and 2 years. Uncorrected distance visual acuity (UDVA), corrected distance visual acuity (CDVA), uncorrected near visual acuity (UNVA), corrected near visual acuity (CNVA), uncorrected intermediate visual acuity (UIVA) with high and low contrast at 63 cm, binocular defocus curve, and reading performance were measured. A patient satisfaction and visual phenomenon questionnaire was administered.

ETDRS chart was used to measure UDVA and CDVA at 4 m and UNVA and CNVA at 35 cm. UIVA under high contrast (100% contrast) and low contrast (10% contrast) at 63 cm was tested using Colenbrander Mixed Contrast Card Set (Precision vision, USA) in all eyes. A cord on each card ensured that the viewing distance was maintained accurately. The intermediate visual acuity at 63 cm was recorded when at least 4 high-contrast or low-contrast targets in each card were identified correctly.

Binocular defocus testing was performed using a 100% contrast ETDRS chart at 4 m under photopic conditions. Manifest refraction was used to designate the zero baselines. A defocus of −5.00 D spherical correction from the corrected distance visual acuity (manifest refraction) was set; the decimal equivalent acuity at this refraction was recorded. Negative spherical power was decreased in 0.50 D increments, with decimal equivalent acuity recorded at each change in correction until only manifest refraction remained. Then, a defocus of +2.00 D spherical correction from the manifest refraction was set and the decimal equivalent acuity was recorded. Positive spherical power was decreased in 0.50 D increments, with decimal equivalent acuity recorded at each change in correction until only manifest refraction remained. The depth of focus was calculated as half the values of visual acuity better than 0.3 LogMAR at different defocus values.

Reading speed was tested at the preferred reading distance using the same text with a 12-point print size and 1.5 line spacing, in accordance with the Radner Reading Charts. Patients were asked to read the same text binocularly as quickly and accurately as possible. The reading time and reading distance were recorded.

A patient satisfaction and visual phenomena questionnaire [11] was administered 2 years postoperatively. Patients rated satisfaction with their vision on a scale from 1 to 10 (1 = incapacitating; 10 = excellent). Patients also rated the incidence of visual phenomena (e.g., glare, halos) on the following scale: 0 = none; 1 = minimal; 2, 3, and 4 = moderate; 5 = severe. Patients' education was assessed from 1 to 5 (1 = primary; 2 = junior; 3 = senior; 4 = college; 5 = university). In addition, patients' reading habit was questioned, including daily reading duration and the percentage of just reading newspaper title.

## 3. Statistical Analysis

All visual acuity values were converted to the logarithm of the minimum angle of resolution (LogMAR) for statistical analysis. Results were expressed as means ± standard deviation. Statistical analysis was performed using SPSS advanced statistical 13.0 software (SPSS Inc.). The Shapiro-Wilk test was used to check normality. The *t*-test or Mann-Whitney *U* test was used to compare the 2 groups. Differences were considered statistically significant when the *P* value was less than 0.05.

## 4. Results

Forty patients had all scheduled examinations. The patients' demographics were shown in Table 1. After cataract extraction and in-the-bag IOL implantation, the pupils of all patients were round and showed good responsiveness to light; there was no case of iris trauma. All IOLs were well centered with no obvious tilt or decentration. Two years postoperatively, spherical diopter was (−0.09 ± 0.40) D versus (−0.02 ± 0.42) D, respectively (range (−0.75~0.75) D versus (−0.50~0.75) D, resp.) (*P* = 0.636). Cylinder diopter was (−0.17 ± 0.49) D versus (−0.22 ± 0.51), respectively (range (−1~0.50) D versus (−1~0.75) D, resp.) (*P* = 0.561).

### 4.1. Visual Acuities

Table 2 shows the mean distance, intermediate, and near visual acuities 2 years after surgery. The UDVA was 20/25 or better in 72.5% of eyes in SN6AD1 group and in 92.5% of eyes in SN60WF group. But the percentage of CDVA 20/25 or better increased to 90% in SN6AD1 group and 100% in SN60WF group. However, there was no statistically significant difference between the 2 groups in mean UDVA (*P* = 0.474) or CDVA (*P* = 0.802).

All eyes in SN6AD1 group and 50% of eyes in SN60WF group achieved a UNVA of 20/40 or better, with 57.5% of eyes in SN6AD1 group and 0 in SN60WF group obtaining the UNVA of 20/25 or better. 85% in SN6AD1 group and 70% of eyes in SN60WF group gained CNVA 20/25 or better. SN6AD1 group acquired significantly better UNVA 0.111 ± 0.897 LogMAR than SN60WF group 0.361 ± 0.798 LogMAR (*P* < 0.001). But statistically significant difference was not found in mean CNVA between the 2 groups (*P* = 0.554).

The SN6AD1 group had statistically significant better UIVA at 63 cm under high and low contrast than SN60WF group (*P* = 0.005 and *P* = 0.011). In SN6AD1 group, 42.5% of eyes and 32.5% of eyes had a 20/25 or better high contrast UIVA and 20/40 or better low contrast UIVA, respectively. In SN60WF group, the percentages were 17.5% and 15%, respectively.

**Figure 1:**
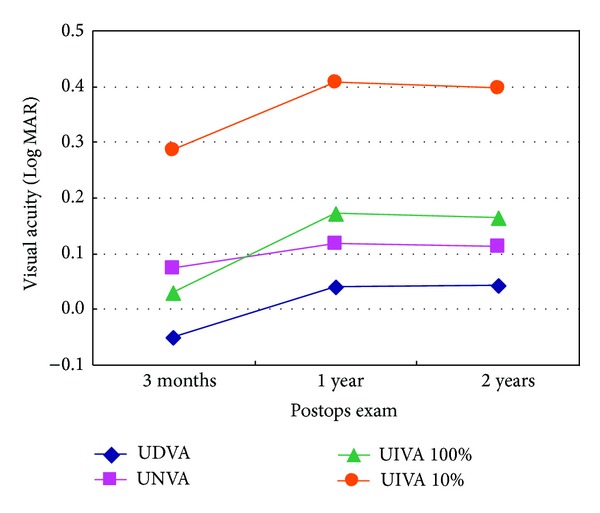
The change of UDVA, UNVA, UIVA (100%), and UIVA (10%) of SN6AD1 MIOL postoperatively.

In terms of SN6AD1 MIOL, 1-year UDVA 0.039 Log MAR and 2-year UDVA 0.041 Log MAR were reduced significantly than postoperative 3-month UDVA −0.051 Log MAR (*P* = 0.000 and *P* = 0.000). Similarly, UNVA, UIVA (100%), and UIVA (10%) were statistically significantly lower than those of postoperative 3-month outcomes respectively (*P* < 0.05), while statistical significance was not found in UDVA, UNVA, UIVA (100%), and UIVA (10%) between 1-year and 2-year postoperatively in SN6AD1 group (*P* > 0.05) (Figure 1).

## 5. Defocus Curves 

**Figure 2:**
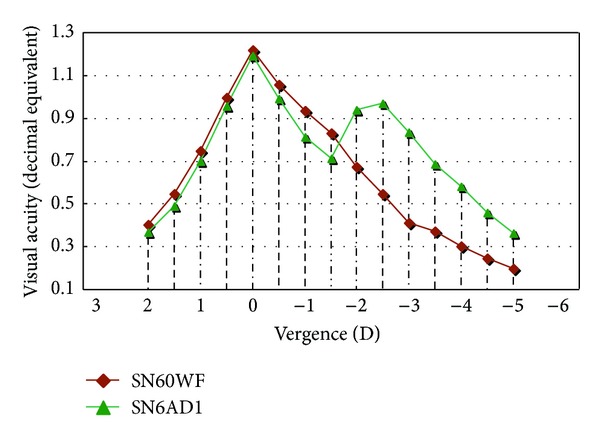
Mean defocus curve for SN6AD1 MOL and SN60WF IOL two years postoperatively. D = diopter.

The mean binocular defocus curves in Figure 2 indicated that SN6AD1 MIOL provided an extended range of visual acuity from near to far. It was shown from the curves that both IOLs achieved approximately the same level of visual acuity at the distance peak (−0.076 LogMar). However, unlike a single distance point at 0 D in SN60WF group, a plateau of visual acuity from the vergence of −2.0 D to −2.5 D was found in SN6AD1 group, the equivalent of 40 cm to 50 cm from the eye. The depth of focus was 5.5 D in SN6AD1 group and 4 D in SN60WF group.

### 5.1. Preferred Reading Distance, Reading Speed, and Reading Habit

The preferred reading distance in both groups was 37.6 cm and 52.8 cm, respectively. SN6AD1 group had a higher speed of reading the fixed text message, (21.40 ± 1.70) s, than SN60WF group, (25.95 ± 2.59) s (*P* < 0.001). Patients' reading habits were shown in Table 3.

### 5.2. Patient Satisfaction and Visual Phenomena Questionnaire

**Figure 3:**
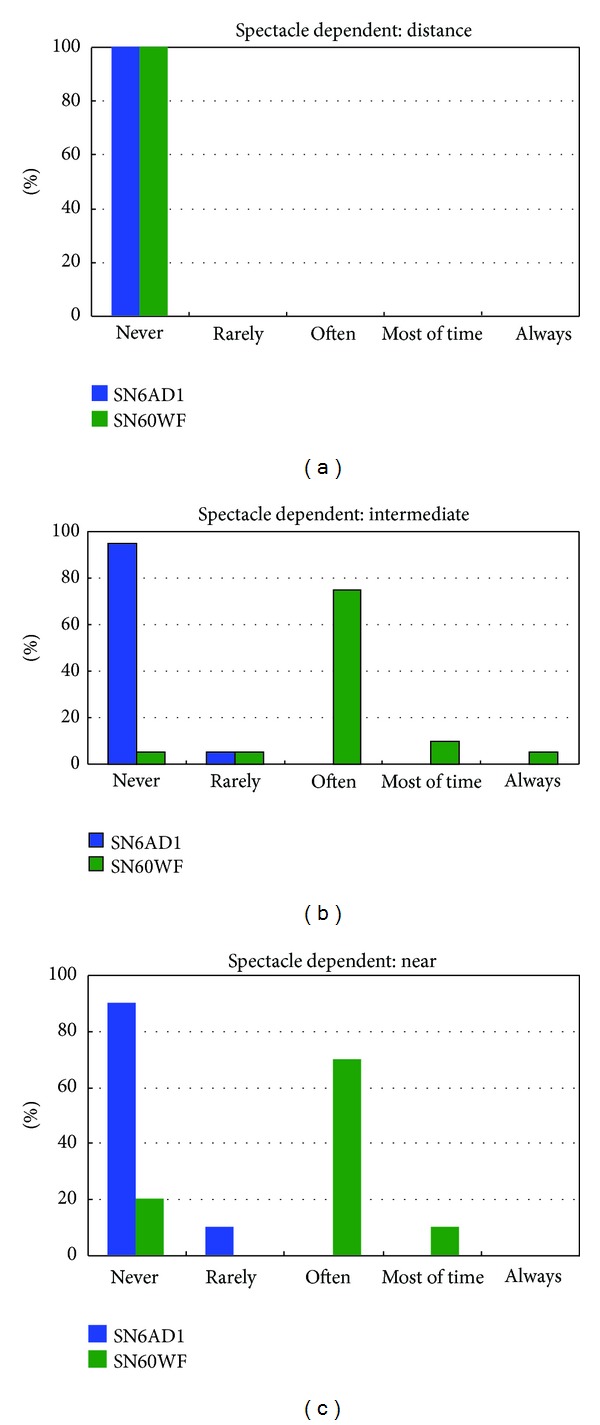
Results of spectacle wearing for distance (a), intermediate (b), and near activities (c).

The quality-of-life questionnaire showed a higher overall vision satisfaction in SN60WF group (*P* = 0.032) (Table 4). But both groups reported no difficulty in all distance activities such as reading, cooking, and shopping (*P* > 0.05) (Table 3). Both groups could perform distance jobs independent of glasses completely (*P* = 1.000), while the near (*P* < 0.001) and intermediate (*P* < 0.05) spectacle independence were higher in SN6AD1 group (Figure 3). Although glare and halo were more severe in SN6AD1 group 0.75 ± 0.85 than SN60WF group 0.15 ± 0.49  (*P* = 0.011), no patient required MIOL removal. Three eyes (7.5%) in SN6AD1 group had moderate glare as a result of posterior capsule opacification (PCO). The symptom glare/halo diminished to a mild level after Nd:YAG laser capsulotomy. SN6AD1 group had a more advanced education level (*P* = 0.038) and higher salary (*P* = 0.022) than SN60WF group (Table 5). SN6AD1 group had higher requirement for reading than that of SN60WF group.

## 6. Discussion

To the best of our knowledge, study of the medium-term or long-term visual performance of SN6AD1 MIOL was not available. In the present study, SN6AD1 MIOL provided better uncorrected/corrected near and distance visual acuity, and uncorrected intermediate visual acuity 2 years postoperatively. SN6AD1 patients had a more comfortable reading distance, faster reading speed, and higher spectacle independence. Although SN6AD1 patients reported a higher incidence of glare and halo, the symptoms improved after Nd:YAG laser capsulotomy.

No comparison with previous studies can be made due to the lack of available long-term data on this type of IOL. However, the outcomes of SN6AD1 group can be compared with the information in our 3-month study for the same participants [16]. UDVA 0.041 LogMar 2 years postoperatively was lower than UDVA −0.051 LogMar 3 months postoperatively. Similarly, UNVA 0.111 LogMar, UIVA (100%) 0.163 LogMar, and UIVA (10%) 0.396 LogMar were inferior to those of 3 months postoperatively. These findings may indicate visual acuities of SN6AD1 MIOL have a decreasing trend over a long-term follow-up. Nevertheless, in the current study, the 2-year UNVA 0.111 LogMar, UIVA (100%) 0.163 LogMar, and UIVA (10%) 0.396 LogMar in SN6AD1 group were statistically significantly better than SN60WF group 0.361 LogMar, 0.260 LogMar, and 0.491 LogMar, respectively, despite that UDVA, CDVA, and CNVA were not statistically different between both groups. In addition, the defocus curves demonstrated SN6AD1 MIOL provided a full range of vision from near to far, with the depth of focus 5.5 D in SN6AD1 group while being 4 D in SN60WF group. This means SN6AD1 MIOL can still provide good visual acuities over a long time after surgery. Regarding SN6AD1 MIOL, 1-year and 2-year UDVA, UNVA, UIVA (100%), and UIVA (10%) were lower than those of 3-month but still much better than SN60WF IOL. It might be a clue that visual acuities of SN6AD1 MIOL would be stable after 1 year postoperatively, which should be verified by long-term study with follow-up for more than 2 years.

One factor in maintaining good full range of vision is the long-term stability of SN6AD1 MIOL and accurate IOL power calculation. Distance visual acuity is affected when decentration of the refractive multifocal IOL exceeds 0.9 mm [19], which expresses the importance of in-the-bag MIOL implantation completely. In fact, it is easy to access the degree of decentration with SN6AD1 MIOL according to its ring structure. In the present study, all MIOLs were implanted in the capsular bag and there was no case of IOL decentration or tilt. It reflected the fact that the perfect surgery was one of the key steps assuring excellent visual function. Moreover, the postoperative refractive error will affect visual acuity [20]. In our study, spherical diopter in SN6AD1 group was (−0.75~0.75) D, and cylinder diopter was (−1~0.50) D. The slight refractive error will aid in obtaining the favorable vision. We believe IOL Master is a reliable instrument for the accurate IOL power calculation.

Although excellent visual acuity is essential to the success of SN6AD1 MIOL, the vision quality it produces is equally important. In the present study, the quality-of-life questionnaire illustrated a relatively lower overall vision satisfaction in SN6AD1 group. It was associated with PCO, patients' different reading habits, education level, and preoperative expectation.

PCO is the most common complication of modern cataract surgery, with an incidence up to 50% at 2 years postoperatively [21]. It has been considered that as MIOLs distribute light to 2 foci, even minor PCO might create symptoms. In our study, 3 eyes (7.5%) complaining about moderate glare in SN6AD1 group required Nd:YAG capsulotomy during the 2-year follow-up, 1 at 9 months, 1 at 15 months, and 1 at 20 months. After Nd:YAG capsulotomy, the moderate glare reduced to mild level and the visual satisfaction scores increased to 7.88 ± 1.11. Considering our study, careful observation of PCO in eyes with SN6AD1 MIOL is important and use of Nd:YAG capsulotomy for the treatment of PCO is a safe method to alleviate undesired visual disturbances and improve visual satisfaction.

Patients' reading habit is another factor influencing visual satisfaction. In our study, all patients with SN6AD1 MIOL read newspapers or magazines daily for average 99 minutes, 95% of whom were reading the main text. By contrast, only 80% patients in SN60WF group had the habit of reading for average 66.25 minutes per one day, and 31% of patients just browsed the headlines. Moreover, the preferred reading distance 37.6 cm in SN6AD1 group was more comfortable for most people, while the distance 52.8 cm in SN60WF group was too far away from eyes. The higher reading speed 21.40 ± 1.70 s in SN6AD1 group partly reflected the better near visual function. However, SN6AD1 patients had to perform more near (including reading) activities, which was in line with their more advanced education level. Although SN60WF patients had no difficulty in performing some near activities revealed in the questionnaire, the simple near tasks likely contributed to the higher satisfaction for SN60WF patients while the stricter requirements and demand for reading in SN6AD1 group would bring down satisfaction.

It is believed that spectacle independence has been positively correlated with overall satisfaction with presbyopia-correcting IOLs [22]. In our study, in accordance with the superior UIVA and UNVA, spectacle independence for intermediate and near vision in SN6AD1 group was higher than SN60WF group.

90% of SN6AD1 patients and 20% of SN60WF patients reported complete spectacle independence for near vision; this changed to 95% and 5% for intermediate vision, respectively. Spectacle freedom and a better vision in daily life will undoubtedly be exciting and are an impetus to high satisfaction. But patients had to pay a lot for the SN6AD1 MIOL and surgery because only part of the cost was refunded by the medical insurance in China. Therefore, they would wish to acquire the best possible visual outcome after operation. The satisfaction would be rated low once the preoperative unrealistic expectation was not achieved. So it is necessary and important for surgeons to try their best efforts to keep patients' preoperative expectation appropriate.

In conclusion, our study showed that SN6AD1 MIOL provided a satisfactory full range of visual acuity, comfortable reading distance, faster reading speed, and high overall spectacle independence 2 years after surgery. One-year and 2-year uncorrected near and intermediate visual acuities of SN6AD1 MIOL were reduced than those of 3-month but they are still much better than SN60WF IOL. SN6AD1 patients' lower vision satisfaction had something to do with PCO, patients' different reading habits, education level, and preoperative improper expectation.

## Figures and Tables

**Table 1:** Patients' characteristics preoperatively.

Characteristics	SN6AD1 group	SN60WF group	*P* value
Number of eyes	40	40	—
Male/female (*n*)	11/9	10/10	—
Age (y)			
Mean ± SD	69.1 ± 9.7	73.3 ± 4.9	0.090
Range	45–81	61–81	
IOL power (D)			
Mean ± SD	19.5 ± 1.0	19.2 ± 1.5	0.339
Range	18–21	17–21	
Axial length (mm)			
Mean ± SD	23.6 ± 0.6	23.7 ± 0.8	0.787
Range	22.5–24.8	22.4–25.3	
Keratometry (D)			
Mean ± SD	44.3 ± 1.9	43.9 ± 1.3	0.474
Range	39.2–47.9	40.5–45.8	

IOL: intraocular lens; D: diopter.

**Table 2:** The distance, intermediate, and near visual acuity (Log⁡MAR) tested at 2 years postoperatively.

	SN6AD1 group	SN60WF group	*Z*	*P*
UDVA	0.041 ± 0.563	0.026 ± 0.850	−0.722	0.474
CDVA	−0.014 ± 0.682	−0.020 ± 0.965	−0.256	0.802
UIVA (100%)	0.163 ± 0.667	0.260 ± 0.702	−2.805	0.005
UIVA (10%)	0.396 ± 0.890	0.491 ± 0.964	−2.536	0.011
UNVA	0.111 ± 0.897	0.361 ± 0.798	−7.032	<0.001
CNVA	0.081 ± 0.959	0.088 ± 0.857	−0.618	0.554

UDVA: uncorrected distance visual acuity; CDVA: corrected distance visual acuity; UIVA: uncorrected intermediate visual acuity; UNVA: corrected near visual acuity.

**Table 3:** Patients' reading habits.

	SN6AD1 group	SN60WF group	*Z*	*P*
Percentage of reading	20/20	16/20		
Daily reading newspaper duration (min)	99.00 ± 45.76	66.25 ± 45.88	−2.096	0.036
Just reading newspaper title (%)	5% (1/20)	31% (5/16)		
Percentage of using mobile phone	20/20	19/20		
Spectacle independence when using phone	20/20	19/19		
Difficulty in reading message	No (*n* = 20)	No (*n* = 19)		
Reading pint size	16.00 ± 1.45	16.21 ± 1.47	−0.457	0.670

**Table 4:** Results of patient satisfaction and visual phenomena questionnaire administered 2 years postoperatively.

Questions	Mean score* ± SD		*P* value
	SN6AD1	SN60WF	
How satisfied are you with your vision?	7.23 ± 1.33	7.95 ± 1.04	0.032
glare/halos	0.75 ± 0.85	0.15 ± 0.49	0.011
How much difficulty do you have with…			
watching TV?	0.05 ± 0.22	0.00 ± 0.00	1.000
reading and near work/activities?	0.00 ± 0.00	0.13 ± 0.34	0.190
cooking?	0.00 ± 0.00	0.00 ± 0.00	1.000
using a cell phone?	0.00 ± 0.00	0.00 ± 0.00	1.000
doing sports?	0.00 ± 0.00	0.00 ± 0.00	1.000
shopping?	0.00 ± 0.00	0.00 ± 0.00	1.000

*Scale for satisfaction with vision ranged from 1 to 10 (1 = incapacitating; 10 = excellent).

Scale for all other questions was 0 = none; 1 = minimal; 2, 3, and 4 = moderate; 5 = severe.

**Table 5:** Patients' education level and job status.

	SN6AD1 group	SN60WF group	*P*
Education level*	3.65 ± 1.27	2.70 ± 1.42	0.038
High school or advanced	80% (16/20)	50% (10/20)	0.047
Working	30% (6/20)	10% (2/20)	0.235
Monthly salary over RMB 5000	80% (16/20)	45% (9/20)	0.022

*Education level: 1 = primary; 2 = junior; 3 = senior; 4 = college; 5 = university.
